# Targeting *EML4-ALK* gene fusion variant 3 in thyroid cancer

**DOI:** 10.1530/ERC-20-0436

**Published:** 2021-04-20

**Authors:** Mehtap Derya Aydemirli, Jaap D H van Eendenburg, Tom van Wezel, Jan Oosting, Willem E Corver, Ellen Kapiteijn, Hans Morreau

**Affiliations:** 1 Department of Pathology, Leiden University Medical Center, Leiden, The Netherlands; 2 Department of Medical Oncology, Leiden University Medical Center, Leiden, The Netherlands

**Keywords:** thyroid cancer, EML4-ALK gene fusion, crizotinib, ceritinib, lorlatinib, targeted therapy, personalized medicine, humans, cancer cell line, cell signaling, ALK, AKT, ERK, STAT

## Abstract

Finding targetable gene fusions can expand the limited treatment options in radioactive iodine-refractory (RAI-r) thyroid cancer. To that end, we established a novel cell line ‘JVE404’ derived from an advanced RAI-r papillary thyroid cancer (PTC) patient, harboring an *EML4*-*ALK* gene fusion variant 3 (v3). Different *EML4-ALK* gene fusions can have different clinical repercussions. JVE404 cells were evaluated for cell viability and cell signaling in response to ALK inhibitors crizotinib, ceritinib and lorlatinib, in parallel to the patient’s treatment. He received, after first-line lenvatinib, crizotinib (Drug Rediscovery Protocol (DRUP) trial), and lorlatinib (compassionate use). *In vitro *treatment with crizotinib or ceritinib decreased viability in JVE404, but most potently and significantly only with lorlatinib. Western blot analysis showed a near total decrease of 99% and 89%, respectively, in pALK and pERK expression levels in JVE404 cells with lorlatinib, in contrast to remaining signal intensities of a half and a third of control, respectively, with crizotinib. The patient had a 6-month lasting stable disease on crizotinib, but progressive disease occurred, including the finding of cerebral metastases, at 8 months. With lorlatinib, partial response, including clinical cerebral activity, was already achieved at 11 weeks’ use and ongoing partial response at 7 months. To our best knowledge, this is the first reported case describing a patient-specific targeted treatment with lorlatinib based on an *EML4*-*ALK* gene fusion v3 in a thyroid cancer patient, and own cancer cell line. Tumor-agnostic targeted therapy may provide valuable treatment options in personalized medicine.

## Introduction

Thyroid cancer is the most common endocrine malignancy and its incidence is on the rise (Siegel *et al.* 2019). Non-medullary thyroid cancer (NMTC) includes differentiated thyroid cancer (DTC), accounting for ~95% of thyroid cancers, with main histologic subtypes papillary (PTC), follicular thyroid carcinoma (FTC) and Hürthle cell carcinoma (HCC). Usually, prognosis is favorable in differentiated thyroid cancer cases with standard therapy including thyroidectomy combined with RAI therapy (Haugen *et al.* 2016). However, a subset may be in or progress to RAI-refractory status which implies a very poor 10-year survival of <10% (Schlumberger *et al.* 2015).

Treatment options in RAI-refractory differentiated thyroid cancer (RAI-rDTC or RRDTC) are limited and include local treatments or registered first-line drugs lenvatinib and sorafenib. When these treatment options have been exhausted, inclusion in trials may sometimes be warranted (Haugen *et al.* 2016). Yet, over the last few years, advanced diagnostics are evolving and their role becomes more pivotal in directing the most suitable patient management choices. The application of therapeutic options across cancer types of various affected organs based on their molecular profiling (a tumor-agnostic approach), facilitates tailored therapy in precision medicine and several basket trials have been initiated and are ongoing for this purpose (ClinicalTrials.gov) (van der Velden *et al.* 2019). However, unique molecular profiles in one type of cancer may potentially render (in)sensitivities to certain drugs that have otherwise proven to be effective in another cancer type. For instance, favorable results with vemurafenib in *BRAF* mutated melanoma vs limited effect in colon cancer (Flaherty *et al.* 2010, Kopetz *et al.* 2015), or panitumumab (EGFR inhibitor) in *RAS* WT colon cancer vs Hürthle cell cancer of the thyroid (Aydemirli *et al.* 2019, Battaglin *et al.* 2019).

Echinoderm microtubule-associated protein-like 4 – anaplastic lymphoma kinase (*EML4*-*ALK*) gene fusions are prevalent in lung cancer and treatment with ALK inhibitors is a part of conventional care (Recondo *et al.* 2018). This set of targetable gene fusions may also present in thyroid cancer (Cancer Genome Atlas Research Network 2014, Demeure *et al.* 2014, Kelly *et al.* 2014, Chou *et al.* 2015, Landa *et al.* 2016, Panebianco *et al.* 2019, van der Tuin *et al.* 2019). *EML4*-*ALK* gene fusions (by the *EML4 *coiled-coil domain that mediates constitutive dimerization) result in constitutive ALK kinase activation (Soda *et al.* 2007, Mano 2008, 2015) and thereby lead to oncogenic signaling via several pathways including phosphatidylinositol 3-kinase (PI3K)/AKT, RAS/extracellular signal-regulated kinase (ERK), Janus kinase/signal transducer and activator of transcription protein (JAK/STAT) (Hallberg & Palmer 2016, Sabir *et al.* 2017). Depending on varying gene fusion points of *EML4*, different *EML4-ALK* fusion variants, of varying lengths, may arise (Sabir *et al.* 2017). For instance, *EML4-ALK* fusion variants 3 and 5 are ‘shorter’ and lack the tandem atypical beta-propeller (TAPE) domain that is present in v1 or v2 (Bayliss *et al.* 2016). Their respective susceptibility to various ALK inhibitors is reported to vary (Heuckmann *et al.* 2012, Sabir *et al.* 2017).

Crizotinib and ceritinib are the first- and a second-generation ALK TKI, respectively, approved for the treatment of patients with ALK-positive advanced non-small cell lung cancer (NSCLC), and lorlatinib is a third-generation ALK TKI approved for previously treated ALK-positive metastatic NSCLC (Recondo *et al.* 2018) (also see https://www.drugs.com/history/xalkori.html, https://www.drugs.com/history/zykadia.html and https://www.drugs.com/history/lorbrena.html). With lorlatinib treatment, the NSCLC patients who harbored *EML4*-*ALK* v3 have been reported to show a longer progression-free survival (PFS) than those harboring *EML4*-*ALK* v1 (Lin *et al.* 2018). With crizotinib, for those with *EML4*-*ALK* fusion variants 1/2/others (i.e. non-v3) longer PFS have been reported than for *EML4*-*ALK* v3 among treated patients (Seo *et al.* 2017, Woo *et al.* 2017, Christopoulos *et al.* 2018). Further studies with crizotinib reported longer PFS for v1 (Yoshida *et al.* 2016), longer PFS for v2 (Li *et al.* 2018), and lower PFS and overall survival (OS) for v3 or v5 than other fusion variants (Su *et al.* 2019). However, several other studies reported no differences in PFS with crizotinib based on the *EML4*-*ALK* fusion variant (Cha *et al.* 2016, Lei *et al.* 2016, Lin *et al.* 2018, 2019, Mitiushkina *et al.* 2018).

All in all, the standard of care regarding ALK TKIs for ALK rearranged NSCLC is rapidly evolving with the elucidation of the complexity, for example, resistance mechanisms, efficacy, CNS-penetrance of various ALK TKIs. In ALK rearranged NSCLC in general, standard first-line treatment is now shifting from crizotinib to second-generation ALK TKIS ceritinib and alectinib (McCusker *et al.* 2019). With crizotinib, its poor CNS-penetrance may account for the fact that CNS metastasis is common among the 70% of patients who failed on this first-generation ALK TKI (Costa *et al.* 2015, McCusker *et al.* 2019). Second- or third-generation ALK TKIs have a higher intracranial activity and may constitute more appropriate ALK TKIs in case of CNS involvement (Zhang *et al.* 2015, McCusker *et al.* 2019).

Thyroid cancer has its own general molecular profile (Pozdeyev *et al.* 2018, van der Tuin *et al.* 2019) with PI3K/AKT and RAS/ERK constituting major signaling pathways involved in thyroid tumorigenesis (Ricarte-Filho *et al.* 2009, Nikiforov 2011, Brehar *et al.* 2013, Xing 2013, Penna *et al.* 2016, Yarchoan *et al.* 2016). Conversely, as various mutations or variants of the *EML4*-*ALK* gene fusion may be present (Sabir *et al.* 2017, Recondo *et al.* 2018), it would be informative to investigate the patient’s treatment response and put real-life outcome into perspective in parallel to a surrogate *in vitro* findings.

At our institution, a patient presented with refractory papillary thyroid cancer (PTC) wherein an* EML4*-*ALK* gene fusion was detected. Hence, after lenvatinib treatment, the patient was included in the Drug Rediscovery Protocol (DRUP) basket trial (NCT02925234) as a candidate for treatment with the ALK tyrosine kinase inhibitor (TKI) crizotinib. From the patient’s papillary thyroid cancer (lymph node metastasis) we derived a primary cell line harboring an *EML4*-*ALK* gene fusion v3. As they share the same essential growth driver due to constitutively activated ALK, we investigated the applicability of ALK inhibitors, such as in conventional therapy of lung cancer harboring *EML4*-*ALK*, to thyroid cancer.

## Materials and methods

### Patient

Lenvatinib was used as standard therapy. Crizotinib was made available within the DRUP trial (ethical approval central committee Dutch Cancer Institute 19 April 2016; Clinical Trials registration October 5, 2016, NCT02925234) (van der Velden *et al.* 2019). Lorlatinib was used within the context of compassionate use. Overall lesion response was evaluated using RECIST 1.1 (Eisenhauer *et al.* 2009). Specimens were handled in compliance with the Code of Conduct for Proper Secondary Use of Human Tissue according to the Federation of Dutch Medical Scientific Societies (Federa) (https://www.federa.org/codes-conduct). The patient was informed about the secondary use of coded residual material for research and had no objections (opt-out policy, exempt from institutional review board approval). Additional informed consent for the publication of anonymized information on clinical data and research on the cancer cell line was obtained from the patient.

### Gene fusion and somatic gene variant screening

Gene fusion and DNA variant analyses were extensively described previously (van der Tuin *et al.* 2019). A fully automated procedure for nucleic acid isolation was used (van Eijk *et al.* 2013). Isolated nucleic acids (DNA and RNA) from resected formalin-fixed paraffin-embedded (FFPE) tumor material were analyzed for the detection of fusion genes using next generation sequencing (NGS) with the Archer® FusionPlex CTL panel (ArcherDX Inc., Boulder, CO, USA). Somatic mutation analysis was performed using NGS with a custom AmpliSeq™ Cancer Hotspot Panel v6 (Thermo Fisher Scientific). Additionally, copy number analysis was performed. Additional TERT-promoter mutation analysis was done using Sanger sequencing, at Macrogen (Amsterdam, the Netherlands). Detected class 5 (pathogenic) and class 4 (likely pathogenic) DNA variations were reported. Within the context of the DRUP trial, metastatic tissue biopsied from the lymph node was analyzed using whole genome sequencing (WGS) (Illumina X10 setup, https://emea.illumina.com/company.html#) at the Hartwig Medical Foundation as described previously by Priestley *et al.* (2019), along with a control blood sample (peripheral blood leukocytes).

### Immunohistochemistry

Immunohistochemistry was performed as previously described (Hermsen *et al.* 2013), with the use of primary antibody: ALK rabbit mAb, 1:100 (Cell Signaling Technology (CST), #3633).

### Compounds

For laboratory assessment, the compounds crizotinib (PF-02341066, Cat. No. S1068, Selleck Chemicals LLC, Houston, TX, USA), ceritinib (LDK378, Cat. No. S7083, Selleck Chemicals LLC, Houston, TX, USA) and lorlatinib (PF-6463922, Cat. No. S7536, Selleck Chemicals LLC, Houston, TX, USA) were prepared as stock solutions of 5 mM in DMSO (J.T. Baker, Avantor Performance Materials Poland S.A., Gliwice, Poland).

### Cell line establishment, DNA/RNA isolation, cell line authentication, cell culture, cell count

Cell line establishment was performed as previously described (Boot *et al.* 2016). Cancer cell line DNA isolation was performed using the NucleoSpin**®** DNA purification kit (Macherey–Nagel GmbH & Co. KG, Düren, Germany) according to the manufacturer’s instruction. Cancer cell line RNA isolation was performed using the NucleoSpin**®** RNA purification kit (Macherey–Nagel GmbH & Co. KG) according to the manufacturer’s instruction. DNA and RNA concentrations were measured using Nanodrop 1000 (Isogen, De Meern, the Netherlands). The cancer cell lines (Table 1) were authenticated by short tandem repeat (STR) profiles (GenePrint^®^ 10 system, Promega). STR profile of the novel cancer cell line JVE404 is as follows: AM: X,Y; CSF1PO: 11,12; D13S317: 9; D16S539: 11,13; D21S11: 31.2,33.2; D5S818: 11,12; D7S820: 10,11; TH01: 9.3; TPOX: 8,1; VWA: 15,17.

**Table 1:** Cancer cell line characteristics.

Cell Line	Sex	Age	Origin	Localization	Gene fusion	Ref.
JVE404	m	61	PTC	LN metas.	*EML4*-*ALK* v3	*
NCI-H2228	f	uk	NSCLC	primary	*EML4*-*ALK* v3 (Koivunen *et al.* 2008), *ALK-PTPN3 *(Jung *et al.* 2012)**	(Phelps *et al.* 1996)
Karpas-299	m	25	TCNHL	primary	*NPM1*-*ALK* (Krumbholz *et al.* 2018)	(Fischer *et al.* 1988)
BHP 2-7	f	uk	PTC	primary	*RET*/PTC1 (Melillo *et al.* 2005, Schweppe *et al.* 2008)	(Ohta *et al.* 1997)

*First described in the present study. **Non-pathogenic gene fusion.

f, female; LN, lymph node; m, male; metas., metastasis; NSCLC, non-small cell lung cancer; PTC, papillary thyroid carcinoma; Ref., references; TCNHL, T cell non-Hodgkin lymphoma; uk, unknown.

Cells were cultured under standard conditions in a humidified atmosphere (5% CO_2_, 95% air, 37°C). Cells of the cell lines JVE404, NCI-H2228 and BHP 2-7 were cultured in DMEM/F-12 medium (Cat. No 11330032, Gibco, Life Technologies) and cells of Karpas-299 in RPMI medium 1640 (Cat. No 52400025, Gibco, Life Technologies). The media were supplemented with penicillin (50 U/mL), streptomycin (50 µg/mL) (Cat. No 15140122, Gibco, Life Technologies) and 10% heat-inactivated fetal bovine serum (Cat. No 758093, Greiner bio-one, Longwood, FL, USA).The cancer cells were tested for mycoplasma using a mycoplasma-specific PCR (van Kuppeveld *et al.* 1993).

Cells were harvested using Hank’s balanced salt solution (HBSS, Sigma–Aldrich) containing 0.125% trypsin (Gibco, Life Technologies) and 0.25 mM EDTA at 37°C for cell passaging.

Cells were counted using a 1:20 dilution of AO-DAPI (solution 18, Cat. No 9103018, Chemometec, Allerød, Denmark), loaded in quadruplicate onto the NC-Slide A8 (Chemometec) and subsequently read out using the automated cell analyzer nucleoCounter NC-250 (Chemometec) with NucleoView NC-250 software (Chemometec).

### Quantitative multiplexed near-infrared fluorescent Western blotting

For the preparation of protein lysates for Western blotting, the cells were cultured until 70 to 80% confluency and treated with DMSO (0.006%), crizotinib (30, 100, 300 nM) or lorlatinib (30, 100, 300 nM) for 2 hours, followed by washing with ice-cold PBS and lysed with Hot-SDS buffer containing PhosSTOP™ (Cat. No 04906837001, Roche Diagnostics) and cOmplete™ (Cat. No 11697498001, Roche Diagnostics). Quantitative multiplexed near-infrared fluorescent Western blotting was performed as previously described (Aydemirli *et al.* 2019). The Bio-Rad DC™ protein assay was used for the determination of protein concentrations, according to the manufacturer’s instructions (Bio-Rad Laboratories, Inc.). For each sample, 20 µg of lysate was mixed with 4× Laemmli sample buffer (Cat. No 1610747, Bio-Rad Laboratories, Inc.) containing βME, heated for 5 min at 100°C and loaded onto a 1.5 mm 10% acrylamide gel in addition to molecular weight markers (92840000 310014776 LI-COR, Lincoln, NE, USA). Electrophoresis was performed at 50 V throughout the gel. The Bio-Rad semi-dry Trans-Blot Turbo Transfer System was used for blotting (Limit 25V, constant 2.5A, 15 min). Blots were washed in 1× TBS, blocked for 1 h in Odyssey blocking buffer (TBS) (Cat. No 92750000, LI-COR, Lincoln, NE) 1:1 with 1× TBS, washed in 1× TBS, incubated overnight at 4°C with primary antibodies in 1× TBS/0.1% Tween-20/5% BSA (Sigma-A9647, Sigma–Aldrich). Blots were washed in 1× TBS/0.1% Tween-20, then incubated for 1 h with secondary antibodies in 1× TBS/0,1% Tween-20/5% BSA protected from light, then washed with 1× TBS. Western blotting experiments were repeated three times. The blots were imaged at high-resolution using the Odyssey infrared imaging system (LI-COR, Lincoln, NE). Image Studio Lite Ver 5.2 software package (LI-COR, Lincoln, NE) was used for image analysis. Signal intensity values were corrected to the loading control (α-Tubulin), followed by percentual calculations against DMSO control, then plotted in bar charts, along with two-way ANOVA followed by Tukey’s* post-hoc* test (statistical significance was considered at *P*  < 0.05), using GraphPad Prism (version 8.0.1 (244) for Windows, GraphPad Software, www.graphpad.com).

### Antibodies

Primary antibodies: anti-α tubulin mouse, 1:50,000 (clone: DM1A, Cat. No. 14450282, eBioscience, San Diego, CA, USA); ALK rabbit mAb, 1:2000 (CST #3633); pALK (Tyr1507) rabbit mAb (CST #14678); Akt mouse mAb, 1:2000 (CST #2920); pAkt (Ser473) rabbit mAb, 1:1000 (CST #9277); Erk 1/2 mouse mAb, 1:1000 (CST #4696); pErk 1/2 (Thr202/Tyr204) rabbit mAb, 1:2000 (CST #4370); STAT3 mouse mAb, 1:1000 (CST #9139); pSTAT3 (Tyr705) mouse mAb, 1:2000 (CST #4113). Secondary antibodies: green-fluorescent goat anti-rabbit IRDye 800CW, 1:10,000 (92632211 LI-COR, Lincoln, NE). Red-fluorescent goat anti-mouse IRDye 680LT, 1:10,000 (92668020 LI-COR, Lincoln, NE).

### Toxicity profiling

Cells were seeded in 96 well cell culture microplates (655090, Greiner Bio-One GmbH, Frickenhausen, Germany) at 10,000 cells per well. Twenty-four hours after seeding, the compounds crizotinib, ceritinib, lorlatinib or DMSO were added to the wells. After 72 h of incubation with the compounds, PrestoBlue™ cell viability reagent (invitrogen by Thermo Fisher Scientific, Life Technologies Corporation) was added to the wells and viability was measured according to the manufacturer’s instructions. The cell viability assessments were performed in quadruplicate and reproduced in two independent experiments. Dose-response curves (expressed as mean values with standard deviation), IC_50_ values and comparison between compounds (RM one-way ANOVA test followed by Tukey’s* post-hoc* test) were determined using GraphPad Prism (version 8.0.1 (244) for Windows, GraphPad Software, www.graphpad.com). Statistical significance was considered at *P*  < 0.05.

## Results

### Patient

**Figure 1:**
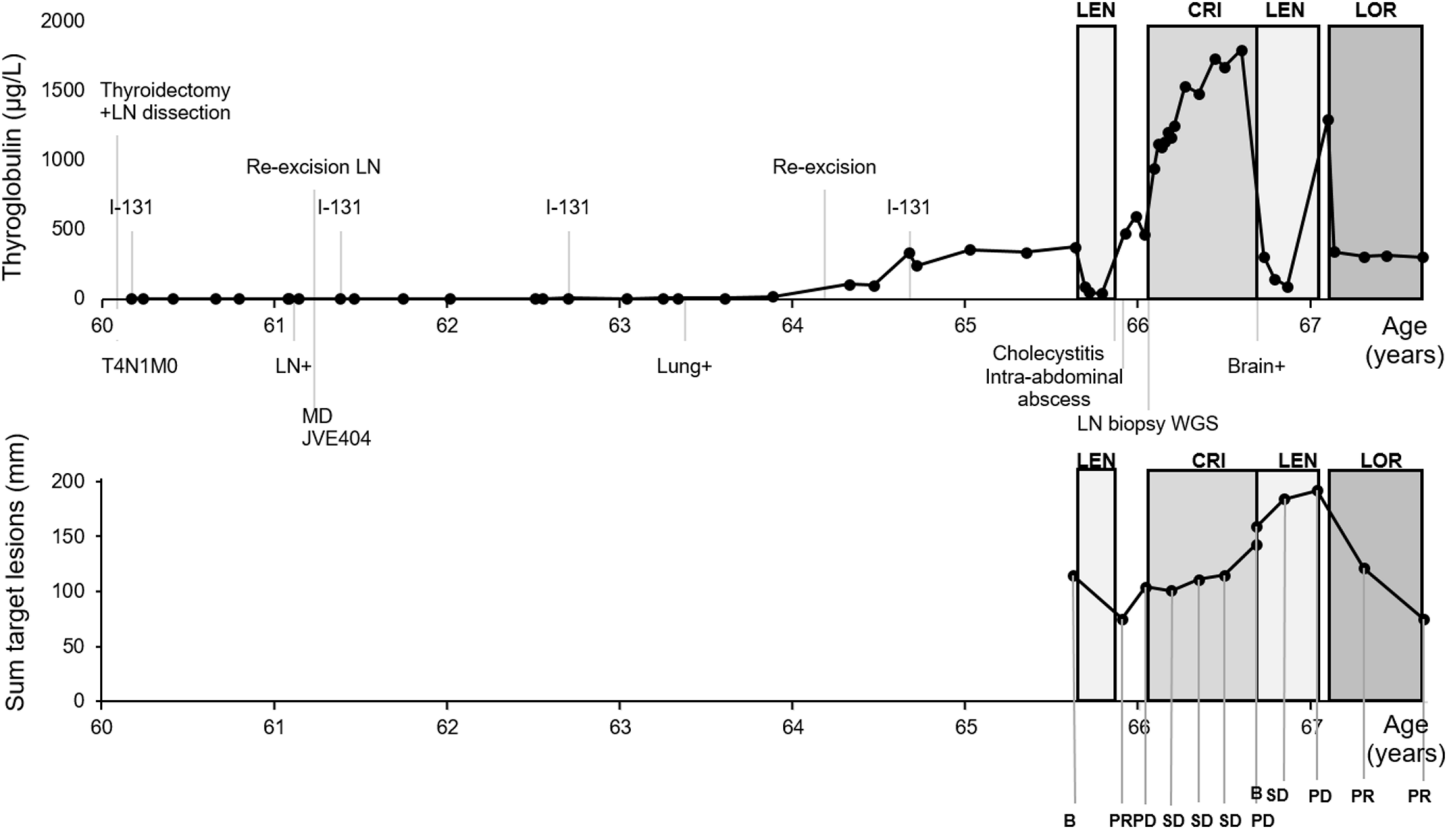
Timeline showing the disease course of our patient since his thyroidectomy, along with thyroglobulin plasma levels (upper line graph) and RECIST evaluations with the sum of the target lesions (lower line graph) during the targeted therapies. The patient received first lenvatinib for two and a half months, then the ALK inhibitor crizotinib within the DRUP trial for 8 months, followed by lenvatinib for four and a half months, and then (until now) the ALK inhibitor lorlatinib for about 7 months as part of compassionate use. MD, molecular diagnostics; LN, lymph node; WGS, whole genome sequencing; LEN, lenvatinib; CRI, crizotinib; LOR, lorlatinib; B, baseline; PR, partial response; SD, stable disease; PD, progressive disease. ‘LN+’, ‘Lung+’, ‘Brain+’, indicates new metastatic spread.

A 60-year-old man without past medical history presented with a palpable thyroid nodule and a swollen lymph node in the neck, suspicious of metastasis. The lymph node was excised and corresponded on histologic examination to a poorly differentiated follicular variant of papillary thyroid carcinoma (FVPTC). Thyroidectomy with lymph node dissection was performed for the T4N1M0-staged partly poorly differentiated papillary thyroid carcinoma with subsequent I-131 therapy, also see Supplementary Fig. 1 (see section on supplementary materials given at the end of this article). Over the next 4 years after initial diagnosis, the I-131 was iterated multiple times to a cumulative dose of 27.7 GBq of RAI (Fig. 1).

A year after the surgery, a re-excision of lymph node metastases in the neck (paratracheal and caudal to the sternocleidomastoid muscle) was performed, corresponding to PTC, partly poorly differentiated. Molecular analysis of tumor tissue showed an *EML4-ALK* gene fusion. *EML4* exon 6, NM_019063.3: *ALK* exon 20, NM_004304.4. Copy number analysis showed homozygous deletion of *CDKN2A* (P16) gene. Also, a *TERT* promoter c.-124C>T (C228T; COSM1716558) variant was detected. No other class 4 or 5 pathogenic variants were detected. Congruently, immunohistochemical analysis of the tumor metastasis in the lymph node showed ALK overexpression in the tumor cells, indicative of the functional nature of this fusion gene. Both the *EML4-ALK* gene fusion and the promoter *TERT* variant were confirmed in the primary tumor.

Despite these therapies, thyroglobulin levels were on the rise and his disease had progressed to radioiodine refractory status. In retrospect, the last RAI dose proved to be unjustified. The patient showed a progression of lymph node metastases (mediastinum, neck, supraclavicular) and pulmonary metastases. No osseous lesions were seen. Recurrent tumor tissue was causing local mechanic compression with focal vascular invasion into the left subclavian vein, which manifested in s.c. edema of the left arm. For decompression, the re-excision of recurrent thyroid tissue along with excision of lymph node metastasis in the neck was performed.

The patient was treated with lenvatinib (the conventional daily dosage of 24 mg) according to local practice with TKI treatment registered for RR-DTC. The patient used lenvatinib for two-and-a-half months till adverse events of acute cholecystitis (due to existent gallbladder stones) and intra-abdominal abscess formation, possibly due to intestinal perforation, emerged, of which relatedness to lenvatinib could not be excluded. Hereupon, lenvatinib was ceased. Due to the relatively short treatment course, response evaluation according to RECIST had not been performed during therapy. However, as a subjective and, in fact, the ostensive measure of clinical benefit, the edema in the patient’s arm had diminished within 2 weeks of lenvatinib therapy, suggestive of response to lenvatinib. When lenvatinib was ceased, partial response was noted on the following CT scan 2 weeks later. However, clinical progression was suspected based on swollenness of the arm and progressive disease (PD) was noted, based on a CT scan according to RECIST 6 weeks later.

Because the somatic *EML4*-*ALK* v3 rearrangement was affirmatively identified in newly acquired biopsy material from a lymph node metastasis using whole genome sequencing, the patient was considered a potential candidate for inclusion in the DRUP trial with ALK as a therapeutic target. Other genetic alterations detected in the biopsy material using WGS were a somatic variant (*MUC6* c. 793G>A, p.(Gly265Ser)). Inactivation of the *CDKN2A* gene encoding for p16 was confirmed. Within the context of the DRUP trial, the first-generation ALK TKI crizotinib was made available to the patient. Two-and- a-half months after the prior lenvatinib regimen, the 66-year-old patient commenced crizotinib treatment with a daily dosage of 2× 250 mg. The first RECIST evaluation showed SD after 2 months use, as well as after 4 and 6 months and no treatment-emergent adverse events had occurred.

After 8 months of crizotinib use, the radiologic evaluation revealed progressive disease on the CT scan of neck/thorax and abdomen and on a CT brain which was made after traumatic injury to the head, multiple cerebral lesions were seen. It is unknown whether these lesions were already present. Hereupon, crizotinib was ceased and lenvatinib was restarted. With lenvatinib use, clinical benefit was noted by the patient with reduced swollenness of palpable lymph nodes and decreasing levels of thyroglobulin. Gastrointestinal adverse events of nausea, vomiting and abdominal pain ensued because of elevation of the left diaphragm, requiring hospital admission but was manageable with analgesics and supportive measures. The first RECIST evaluation showed SD after 2 months of lenvatinib treatment. However, the evaluation after four-and-a-half months showed PD on the CT scan of the neck/thorax and abdomen and the progression of multiple cerebral metastases, whereupon lenvatinib was stopped.

**Figure 2:**
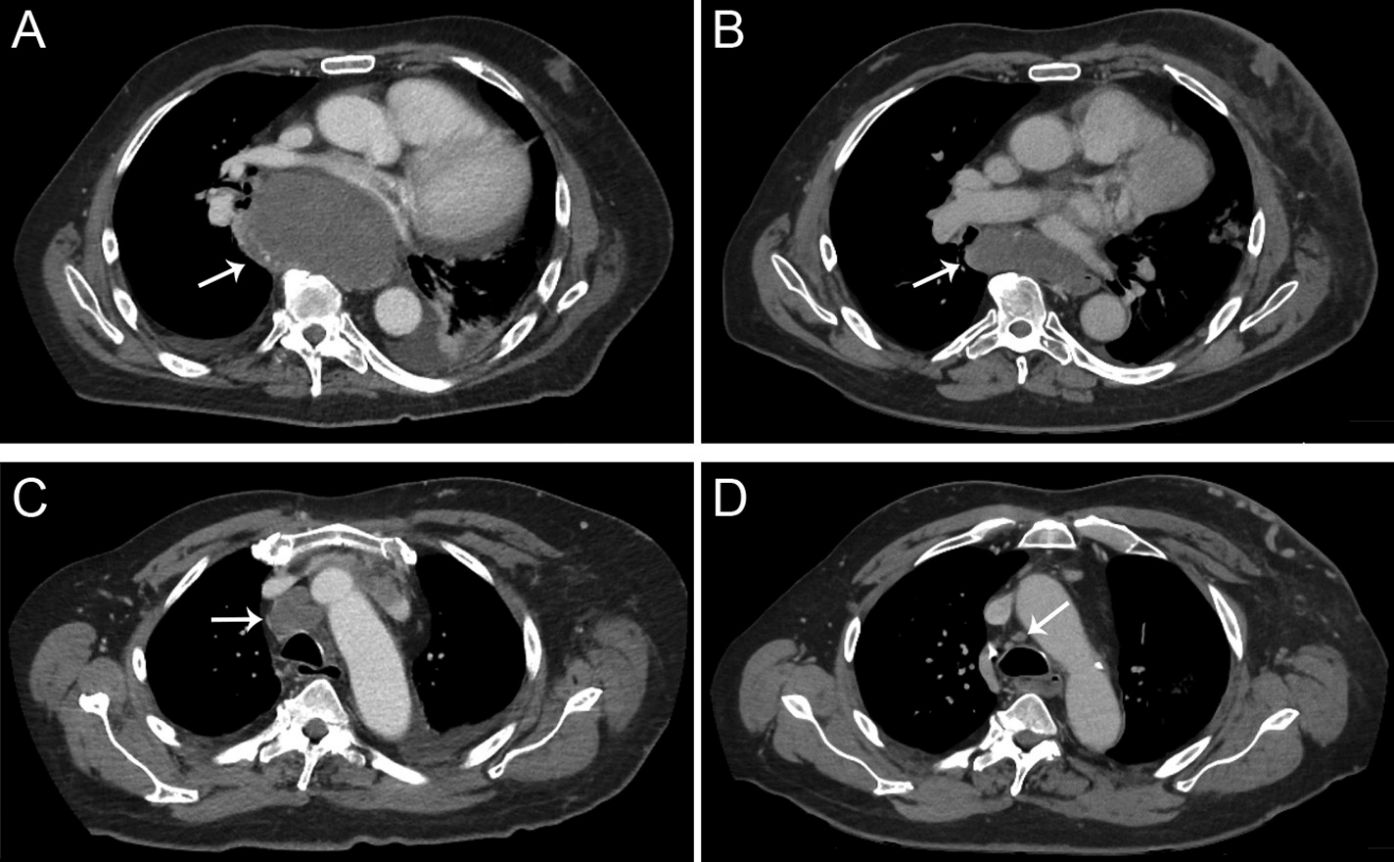
CT scan impressions of the (ongoing) partial response (according to RECIST) on 7 months of lorlatinib treatment. The sum of four target lesions has decreased from 192 mm at baseline to 75 mm; two of these target lesions are shown in the figure. A subcarinal lymph node metastasis reduced from 64 mm at baseline (A) to 28 mm after 7 months lorlatinib (B) in diameter. A pretracheal lymph node metastasis showed a reduced diameter from 29 mm at baseline (C) to 8 mm after 7 months lorlatinib treatment (D).

Two weeks later, within the context of compassionate use and on basis of the tumor cell line experiments described below, the patient started with the third-generation ALK TKI lorlatinib (100 mg per day). Within 2 weeks, thyroglobulin levels dropped by about 75%. After 11 weeks of lorlatinib therapy, the CT scan showed partial response (PR), with a 37% decrease in the sum of the target lesions. Also after 7 months of initiation with lorlatinib treatment, an ongoing PR was determined with a 38% decrease in the sum of the target lesions as compared with the previous CT scan (61% decrease from baseline), also see Figs 1 and 2. Two of three cerebral foci also decreased in maximum diameter (from 9 to 6 mm, and 10 mm to indiscernible, respectively), while one focus showed growth (from 8 to 12 mm). No toxicity was reported with lorlatinib. The patient is currently alive, he has been scheduled for stereotactic radiotherapy for the cerebral lesions and treatment with lorlatinib is ongoing.

### Matching novel thyroid cancer cell line

JVE404 (Supplementary Fig. 2), the PTC-derived cell line harboring the *EML4*-*ALK* gene fusion variant 3 has been established from the patient’s lymph node at age 61 that was resected 1 year after thyroidectomy. Localization of the known papillary thyroid cancer was present in the lymph node but with poorly differentiated parts and frequent mitotic figures. STR profiles of the novel cancer cell line JVE404 matched with the original metastatic material localized in the lymph node. Immunohistochemical analyses of the cancer cell line (passage number 23) also showed ALK expression (ALK-positive) and confirmed thyroidal origin (TTF1 positive, PAX8 positive, thyroglobulin negative) (Baloch *et al.* 2018). The absence of thyroglobulin staining may comply with poorly differentiated thyroid cancer (PDTC) (Baloch *et al.* 2018) or with loss of thyroglobulin expression in cell culture in absence of TSH (Bravo *et al.* 2013).

### Toxicity profiling

As the specific variants of the *EML4-ALK* gene fusion may be associated with higher sensitivity or resistance to certain types of ALK inhibitors, additional clinically used 2nd and 3rd generation ALK TKIs (ceritinib and lorlatinib, respectively) were tested in comparison to crizotinib (1st generation ALK TKI) that was administered to the patient.

**Figure 3 fig3:**
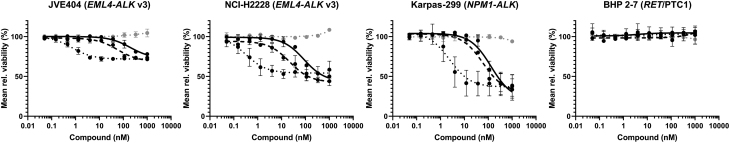
Dose–response curves. In the graphs, the mean relative viability of JVE404, NCI-H2228, Karpas-299 and BHP 2-7 cancer cells are shown for increasing concentrations of DMSO control (grey dot line) and treatment with three ALK TKIs (black lines): crizotinib (solid line), ceritinib (dash line), lorlatinib (dot line).

Treatment with crizotinib, ceritinib or lorlatinib led to decreased viability in JVE404, H2228 and Karpas-299 (Fig. 3 and[Fig fig3] Table 2). As shown in the dose-response curves, the cancer cell lines had a higher sensitivity to lorlatinib as compared to crizotinib. In JVE404, the inhibitory effect of only lorlatinib was significantly superior compared to control (*P* = 0.0022), while crizotinib (*P* = 0.239) or ceritinib (*P* = 0.0624) were not. Further, in JVE404, the inhibitory effect of lorlatinib was significantly higher compared to crizotinib (*P* = 0.0097) and of ceritinib compared to crizotinib (*P* = 0.0153). In NCI-H2228, the inhibitory effect of lorlatinib (*P* = 0.0006) and of ceritinib (*P* = 0.015), respectively, was significantly superior compared to control, while crizotinib (*P* = 0.1217) was not. In NCI-H2228, both lorlatinib and ceritinib, respectively, were significantly superior compared to crizotinib. In Karpas-299, only lorlatinib showed significantly superior inhibition compared to control (*P* = 0.0247). The DMSO control appeared to exert no substantial toxicity. BHP 2-7, the negative control cell line harboring no ALK target, showed no substantial change in response to the compounds on cell viability.

**Table 2:** IC_50_ values, with 95% CI, for the ALK TKIs in the cancer cell lines.

	JVE404	NCI-H2228	Karpas-299
Crizotinib	196.4 nM, 95% CI: 113.8–350.2	86.8 nM, 95% CI: 56.1–134.7	131.3 nM, 95% CI: 77.4–228.7
Ceritinib	58.1 nM, 95% CI: 34.7–97.0	21.6 nM, 95% CI: 12.3–37.4	59.9 nM, 95% CI: 36.1–99.9
Lorlatinib	0.718 nM, 95% CI: 0.483–1.069	0.322 nM, 95% CI: 0.159–0.672	2.399 nM, 95% CI: 1.104–5.310

IC_50_, half maximal inhibitory concentration.

### Western blot analysis

**Figure 4 fig4:**
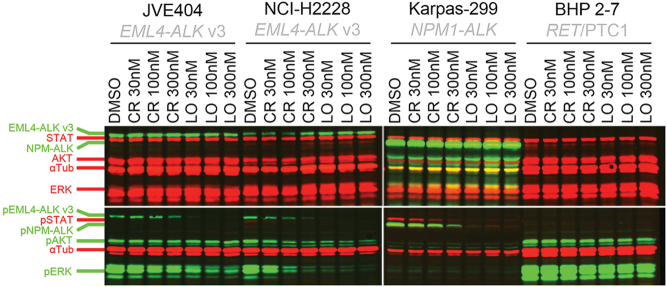
Quantitative, multiplexed near-infrared fluorescent Western blotting. In the Western blot, the expression of proteins and phosphorylated proteins ((p)ALK as the fusion protein of EML4 or NPM1 with conformable molecular sizes, (p)STAT, (p)AKT, (p)ERK and household protein control α-Tubulin) for the cancer cell lines JVE404, NCI-H2228, BHP 2-7 and Karpas-299 are shown in the treated conditions of DMSO control, crizotinib (30, 100, 300 nM) and lorlatinib (30, 100, 300 nM), respectively. αTub, α-Tubulin; CR, crizotinib; LO, lorlatinib.

Western blot analysis (Fig. 4) showed a gradual decrease in pALK and pERK expression upon 30, 100, 300 nM crizotinib on the *EML*-*ALK* v3 harboring JVE404 cells (pALK +2, −8, −49%, respectively, and pERK −35, −63, −68%, respectively), but less pronounced than on NCI-H2228 (lung cancer cell line, also characterized by *EML4*-*ALK* v3) (pALK −35, −59, −80%, respectively, and pERK −58, −89, −96%, respectively) (also see Supplementary Figs 3 and 4). Comparatively, the impact on protein expression levels of phosphorylated ALK and downstream effectors was higher with lorlatinib (JVE404: pALK −93, −97, −98%, respectively, and pERK −79, −89, −88%, respectively; NCI-H2228: pALK −94, −98, −99%, respectively, and pERK −97, −97, −98%, respectively).

Interestingly, despite these significantly reduced expression levels in JVE404 of pALK, up to −49 (*P* = 0.004) and -98% (*P*  < 0.0001), upon increasing crizotinib dose or lorlatinib treatment, respectively, the treatment did not show a strong change in expression levels of pAKT (about −30 and −22%, respectively, not significant) in JVE404. The latter holds true also in contrast to pAKT in NCI-H2228 cells that showed significantly reduced expression (up to −73 and −79% with crizotinib and lorlatinib, respectively, *P*  < 0.0001) levels upon treatment. However, compared to JVE404, the baseline expression level of pAKT was ~20% higher in NCI-H2228 and highest (>16×) in BHP 2-7 cells (Fig. 4,4also see Supplementary Figs 5 and 6). NCI-H2228 cells seemed to express pSTAT, albeit at low levels, but nevertheless ~3× more than JVE404 (pSTAT expression not reliably distinguishable) (also see Supplementary Fig. 3C).

In the control cells Karpas-299 (harboring *NPM1*-*ALK*) ALK was relatively overexpressed and pALK and pSTAT expression levels showed a decrease (up to −84 and −86%, respectively, *P*  < 0.0001) upon crizotinib and even more upon lorlatinib (up to −98 and −94%, respectively, *P*  < 0.0001). In Karpas-299, expression levels of pAKT and pERK were not reliably distinguishable. In the control cells BHP 2-7 (harboring *RET*/PTC1) ALK was not present indeed, STAT was present, but pSTAT expression was not distinguishable. In BHP 2-7, the levels of pAKT fluctuated for the various treatment conditions with DMSO and ALK TKIs, while remaining overexpressed. The levels of pERK decreased upon treatment in BHP 2-7 cells, however, still overexpressed, unlike JVE404 or NCI-H2228.

## Discussion

We present a patient with metastatic refractory thyroid cancer harboring *EML4*-*ALK* gene fusion variant 3. This patient was treated with the ALK inhibitor lorlatinib after matching tumor cell line data showed that lorlatinib was a more potent drug than the already administered crizotinib. Apart from inhibiting EML-ALK v3 better than crizotinib, lorlatinib also exhibited CNS-activity.

The novel and unique patient’s cancer cell line JVE404 showed response, in terms of viability and reduction in cell signaling effectors, to ALK inhibitors *in vitro*. Moreover, the *in vitro* impact of targeting constitutively activated ALK in the context of a variant 3 of the *EML4*-*ALK* gene fusion was more pronounced with lorlatinib (highly significant compared to control and lowest IC_50_ value) than for crizotinib (not significant compared to control) or ceritinib (significance and half-maximal inhibitory value inferior to lorlatinib; not significant compared to control in JVE404, but significant in NCI-H2228). The latter corroborates previous studies in lung cancer (Zou *et al.* 2015, Seo *et al.* 2017, Woo *et al.* 2017, Lin *et al.* 2018).

The presented approach of investigating single cases and cell lines may serve as a rationale to substantiate the use of tested drugs in thyroid cancer by evaluating cell signaling mechanisms combined with analysis of cell viability. Although valuable, this is a very exceptional approach as even only the establishment of a cancer cell line takes a large amount of time and effort and is rarely successful. Demeure * et al.* also described a papillary thyroid cancer patient carrying an *EML4-ALK *v1 gene fusion with stable disease on 6 months’ crizotinib treatment (Demeure *et al.* 2014). To the best of our knowledge, our report presents the first case of an advanced thyroid cancer patient carrying an *EML4-ALK *gene fusion v3 who was treated with lorlatinib. The present study also demonstrates the applicability of a tumor-agnostic approach, whilst carefully taking note of various considerations. Although, this is not yet approved common practice for every druggable mutation that is clinically confirmed in another cancer type.

With tissue-agnostic targeted therapy approaches, such as in the context of basket trials or in the context of compassionate use, it is interesting to investigate the intended impact against the targeted tumor’s own background.

Presumably, the differences in expression levels of the phosphorylated proteins as shown in the Western blot analysis may be indicative of relative insensitivity to the compound, as shown by varying IC_50_ values. It may also be a reflection of general cell signaling characteristics inherent to the cell line with its specific gene fusions or gene variants or to the organ it was derived from. For instance, our patient’s PTC-derived cell line showed pAKT protein expression that remained relatively unaffected (decreased signal intensity of ~20%, not significant), in contrast to a near total 98–88% decrease of pALK and pERK expression, and in contrast to the lung cancer-derived cell line NCI-H2228 (also harboring *EML4-ALK* v3) that showed a decrease of ~80%. Major signaling pathways involved in thyroid tumorigenesis include the MAPK and the PI3K/AKT signaling pathways (Nikiforov 2011, Xing 2013). Moreover, in PTC, pAKT is upregulated (Miyakawa *et al.* 2003, Vasko 2004, Faustino *et al.* 2012, Matson *et al.* 2017) and its nuclear expression has been associated with metastases (Tavares *et al.* 2018). Therefore, in general, AKT seems an interesting target of therapy. On the other hand, it has been shown by a previous study that depletion of EML4-ALK v1 or v3 suppressed pERK and pSTAT3, but not pAKT in non-transformed mouse fibroblast cells and in the lung cancer cell line H3122 (harboring *EML4-ALK* v1); therefore suggesting activation of ERK and STAT3 signaling by such gene fusions but not of the PI3K-AKT pathway (Takezawa *et al.* 2011). Moreover, in comparison to two other thyroid cancer cell lines with known constitutively activated AKT (Aydemirli *et al.* 2019) (being XTC.UC1 cells derived from Hürthle cell cancer and BHP 2-7 cells derived from PTC harboring *RET*/PTC1), baseline pAKT expression levels in JVE404 were ~94% lower (as shown in Supplementary Figs 5 and 6). Also baseline levels of pSTAT in JVE404 were about a third of the expression in NCI-H2228 and subtly discernible, and in these two cell lines, about 97% lower compared to Karpas-299. However, STAT3 activation has been shown to be strongly implicated in lymphomagenesis mediated by *NPM-ALK* (Chiarle *et al.* 2005). These considerations, taken altogether, suggest that in JVE404 predominantly the MAPK pathway is upregulated by the *EML4-ALK* gene fusion v3, as reflected by highly activated ERK that could be brought down concurrently with pALK, upon treatment with ALK inhibitors. This appears to be in line with previous findings of dependence on MAPK in lung adenocarcinoma harboring *EML4-ALK* (Takezawa *et al.* 2011, Hrustanovic *et al.* 2015).

In terms of effectiveness and toxicity, targeted therapy might potentially be more advantageous when an essential growth driver is aimed over a multi-targeted therapy approach (Mano 2015). Kohler * et al.* showed that constitutively activated ALK induced metastatic, poorly differentiated thyroid cancer (PDTC) in mice; therefore, it appears to be a driver of thyroid carcinogenesis (Kohler *et al.* 2019). Moreover, ALK-driven thyroid cancers seem to be associated with solid/trabecular architecture and an increased mitotic rate in PDTC or anaplastic thyroid carcinoma (ATC) (Hamatani *et al.* 2012, Godbert *et al.* 2015, Kohler *et al.* 2019). Detailed characterization of ALK-driven thyroid cancer has recently been described elsewhere, with infiltrative (FV)PTC as the most typical morphology (Panebianco *et al.* 2019). For instance, in the ATC case study by Godbert and colleagues (Godbert *et al.* 2015), a patient is reported with an *ALK* rearrangement in both its well-differentiated part as well as anaplastic part of the tumor. Also, the presence of the ALK rearrangement in both components would favor it to constitute an early carcinogenic driver event, in addition to the excellent response to crizotinib in their patient (Godbert *et al.* 2015). The histologic examination concerning our patient case had revealed FVPTC in a lymph node metastasis, partly poorly differentiated PTC in the primary tumor (also see Supplementary Fig. 1), and metastases with poorly differentiated areas. This seems to fit the range of previously observed morphologies in ALK-driven thyroid cancer.

Furthermore, the impact of effectively targeting essential growth drivers might probably prove to be even more relevant in RAI-rDTC in particular, regarding the potential of possibly inducing redifferentiation and contingent regain of iodine uptake (Buffet *et al.* 2020). The latter, in case it would supervene from the treatment with the drug, may facilitate radioactive-iodine therapy in the patient, and thereby, potentially raising the chances for a cure rather than a prolongation of the progression-free interval (Buffet *et al.* 2020). However, due to practical reasons, we could not investigate this.

Another interesting point of discussion is that a CDK4/6 inhibitor, currently available in the DRUP study (van der Velden *et al.* 2019), constitutes an additional (future) option for targeted therapy aiming for the homozygous *CDKN2A* deletion in our patient. Moreover, even in the absence of this deletion, a synergistic *in vitro* activity of the combination of an ALK inhibitor with a CDK inhibitor in neuroblastoma was reported in the literature (Wood *et al.* 2017). However, this dual therapy was not tested in our experiments or clinically applicable.

Regarding the general toxicity profile; that of available multi-targeted TKIs registered for RAI-rDTC, lenvatinib or sorafenib (Yu *et al.* 2019), may differ from that of lorlatinib (Bauer *et al.* 2019) or crizotinib (Hou *et al.* 2019), as is also illustrated by the patient. Also, the patient described in the current study developed CNS metastasis after treatment with crizotinib failed, as may be seen in a subset of patients who failed on crizotinib treatment due to its poor CNS-penetrance (Costa *et al.* 2015, McCusker *et al.* 2019). In that regard, higher generation ALK TKIs, such as lorlatinib, may be considered in case of CNS metastasis (McCusker *et al.* 2019).

## Conclusion

In this study, ALK targeted therapy with crizotinib and lorlatinib in a thyroid cancer patient harboring an *EML4*-*ALK* gene fusion v3, is illustrated. Additionally, analyses of cell viability and cell-signaling protein molecules have been performed on the novel papillary thyroid cancer cell line harboring an *EML4*-*ALK* v3 derived from the patient’s tumor. Our findings corroborate that also in thyroid cancer with *EML4*-*ALK* v3, targeting ALK appears feasible. We observed clinical activity and *in vitro* impact on viability and downstream signaling in response to the 1st generation ALK TKI crizotinib, although stable disease lasted 6 months and progressive disease included the finding of cerebral metastases at 8 months, but higher sensitivity and clinical partial response with CNS activity to the 3rd generation ALK TKI lorlatinib. With the increasing application of techniques as NGS in daily clinical practice, it seems valuable to recognize molecularly targetable drivers, when treatment options are limited, and even more so in refractory thyroid cancer.

## Supplementary Material

Supplemental Figure S1

Supplemental Figure S2

Supplemental Figure S3

Supplemental Figure S4

Supplemental Figure S5

Supplemental Figure S6

## Declaration of interest

E K is in Advisory boards BMS, Novartis, Roche, Merck, Amgen, Pierre-Fabre, EISAI, Bayer, Genzyme-Sanofi and has received research grants from Novartis and BMS. H M is an advisor in GenomeScan, Leiden, The Netherlands. The other authors declare no conflict of interest.

## Funding

This work did not receive any specific grant from any funding agency in the public, commercial, or not-for-profit sector.

## Author contribution statement

M D A was involved in setting up, performing laboratory experiments and analysis (toxicity profiling, Western blotting, DNA/RNA isolation; cell culturing and cell counting involved), drafting the manuscript, preparation figures, tables and supplementary. J v E was involved in the establishment of cancer cell line JVE404, cell culture. T v W is a registered molecular scientist in pathology and was involved in setting up gene fusion analysis, critical evaluation of the manuscript. J O advised on statistical analysis. W E C was involved in supervising the design of toxicity profiling, supervising Western blot analysis, critical evaluation of the manuscript. E K is an attending medical oncologist in charge of the recurrent endocrine cancer patient care and a contact person for DRUP trial and compassionate use program pharmaceutical company(ies), critical evaluation of the manuscript. H M is an attending pathologist and registered molecular scientist in pathology and was involved in study concept and rationale, supervising data analysis, drafting the manuscript and final evaluation of the manuscript. Ellen Kapiteijn and Hans Morreau were the shared last authors.

## References

[bib1] Aydemirli MD Corver W Beuk R Roepman P Solleveld-Westerink N Van Wezel T Kapiteijn E Morreau H 2019 Targeted treatment options of recurrent radioactive iodine refractory Hürthle cell cancer. Cancers 11 1185. (10.3390/cancers11081185) PMC6721552 31443247

[bib2] Baloch Z Mete O Asa SL 2018 Immunohistochemical biomarkers in thyroid pathology. Endocrine Pathology 29 91–112. (10.1007/s12022-018-9532-9) 29744727

[bib3] Battaglin F Puccini A Ahcene Djaballah S Lenz HJ 2019 The impact of panitumumab treatment on survival and quality of life in patients with RAS wild-type metastatic colorectal cancer. Cancer Management and Research 11 5911–5924. (10.2147/CMAR.S186042) 31388315 PMC6607986

[bib4] Bauer TM Felip E Solomon BJ Thurm H Peltz G Chioda MD Shaw AT 2019 Clinical management of adverse events associated with lorlatinib. Oncologist 24 1103–1110. (10.1634/theoncologist.2018-0380) 30890623 PMC6693708

[bib5] Bayliss R Choi J Fennell DA Fry AM Richards MW 2016 Molecular mechanisms that underpin EML4-ALK driven cancers and their response to targeted drugs. Cellular and Molecular Life Sciences 73 1209–1224. (10.1007/s00018-015-2117-6) 26755435 PMC4761370

[bib6] Boot A Van Eendenburg J Crobach S Ruano D Speetjens F Calame J Oosting J Morreau H Van Wezel T 2016 Characterization of novel low passage primary and metastatic colorectal cancer cell lines. Oncotarget 7 14499–14509. (10.18632/oncotarget.7391) 26894854 PMC4924731

[bib7] Bravo SB Garcia-Rendueles ME Garcia-Rendueles AR Rodrigues JS Perez-Romero S Garcia-Lavandeira M Suarez-Fariña M Barreiro F Czarnocka B Senra A 2013 Humanized medium (h7H) allows long-term primary follicular thyroid cultures from human normal thyroid, benign neoplasm, and cancer. Journal of Clinical Endocrinology and Metabolism 98 2431–2441. (10.1210/jc.2012-3812) 23539720

[bib8] Brehar AC Brehar FM Bulgar AC Dumitrache C 2013 Genetic and epigenetic alterations in differentiated thyroid carcinoma. Journal of Medicine and Life 6 403–408. 24868250 PMC4034295

[bib9] Buffet C Wassermann J Hecht F Leenhardt L Dupuy C Groussin L Lussey-Lepoutre C 2020 Redifferentiation of radioiodine-refractory thyroid cancers. Endocrine-Related Cancer 27 R113–R132. (10.1530/ERC-19-0491) 32191916

[bib10] Cancer Genome Atlas Research Network 2014 Integrated genomic characterization of papillary thyroid carcinoma. Cell 159 676–690. (10.1016/j.cell.2014.09.050) 25417114 PMC4243044

[bib11] Cha YJ Kim HR Shim HS 2016 Clinical outcomes in ALK-rearranged lung adenocarcinomas according to ALK fusion variants. Journal of Translational Medicine 14 296. (10.1186/s12967-016-1061-z) PMC5069800 27756333

[bib12] Chiarle R Simmons WJ Cai H Dhall G Zamo A Raz R Karras JG Levy DE Inghirami G 2005 Stat3 is required for ALK-mediated lymphomagenesis and provides a possible therapeutic target. Nature Medicine 11 623–629. (10.1038/nm1249) 15895073

[bib13] Chou A Fraser S Toon CW Clarkson A Sioson L Farzin M Cussigh C Aniss A O’Neill C Watson N 2015 A detailed clinicopathologic study of ALK-translocated papillary thyroid carcinoma. American Journal of Surgical Pathology 39 652–659. (10.1097/PAS.0000000000000368) PMC4415964 25501013

[bib14] Christopoulos P Endris V Bozorgmehr F Elsayed M Kirchner M Ristau J Buchhalter I Penzel R Herth FJ Heussel CP 2018 EML4-ALK fusion variant V3 is a high-risk feature conferring accelerated metastatic spread, early treatment failure and worse overall survival in ALK(+) non-small cell lung cancer. International Journal of Cancer 142 2589–2598. (10.1002/ijc.31275) 29363116

[bib16] Costa DB Shaw AT Ou SH Solomon BJ Riely GJ Ahn MJ Zhou C Shreeve SM Selaru P Polli A 2015 Clinical experience with crizotinib in patients with advanced ALK-rearranged non-small-cell lung cancer and brain metastases. Journal of Clinical Oncology 33 1881–1888. (10.1200/JCO.2014.59.0539) 25624436 PMC4451171

[bib17] Demeure MJ Aziz M Rosenberg R Gurley SD Bussey KJ Carpten JD 2014 Whole-genome sequencing of an aggressive BRAF wild-type papillary thyroid cancer identified EML4-ALK translocation as a therapeutic target. World Journal of Surgery 38 1296–1305. (10.1007/s00268-014-2485-3) 24633422

[bib18] Eisenhauer EA Therasse P Bogaerts J Schwartz LH Sargent D Ford R Dancey J Arbuck S Gwyther S Mooney M 2009 New response evaluation criteria in solid tumours: revised RECIST guideline (version 1.1). European Journal of Cancer 45 228–247. (10.1016/j.ejca.2008.10.026) 19097774

[bib19] Faustino A Couto JP Populo H Rocha AS Pardal F Cameselle-Teijeiro JM Lopes JM Sobrinho-Simoes M Soares P 2012 mTOR pathway overactivation in BRAF mutated papillary thyroid carcinoma. Journal of Clinical Endocrinology and Metabolism 97 E1139–E1149. (10.1210/jc.2011-2748) 22549934

[bib20] Fischer P Nacheva E Mason DY Sherrington PD Hoyle C Hayhoe FG Karpas A 1988 A Ki-1 (CD30)-positive human cell line (Karpas 299) established from a high-grade non-Hodgkin’s lymphoma, showing a 2;5 translocation and rearrangement of the T-cell receptor beta-chain gene. Blood 72 234–240. 3260522

[bib21] Flaherty KT Puzanov I Kim KB Ribas A Mcarthur GA Sosman JA O’Dwyer PJ Lee RJ Grippo JF Nolop K 2010 Inhibition of mutated, activated BRAF in metastatic melanoma. New England Journal of Medicine 363 809–819. (10.1056/NEJMoa1002011) PMC3724529 20818844

[bib22] Godbert Y Henriques De Figueiredo B Bonichon F Chibon F Hostein I Perot G Dupin C Daubech A Belleannee G Gros A 2015 Remarkable response to crizotinib in woman with anaplastic lymphoma kinase-rearranged anaplastic thyroid carcinoma. Journal of Clinical Oncology 33 e84–e87. (10.1200/JCO.2013.49.6596) 24687827

[bib23] Hallberg B Palmer RH 2016 The role of the ALK receptor in cancer biology. Annals of Oncology 27 (Supplement 3) iii4–iii15. (10.1093/annonc/mdw301) 27573755

[bib24] Hamatani K Mukai M Takahashi K Hayashi Y Nakachi K Kusunoki Y 2012 Rearranged anaplastic lymphoma kinase (ALK) gene in adult-onset papillary thyroid cancer amongst atomic bomb survivors. Thyroid 22 1153–1159. (10.1089/thy.2011.0511) 23050789 PMC3487115

[bib25] Haugen BR Alexander EK Bible KC Doherty GM Mandel SJ Nikiforov YE Pacini F Randolph GW Sawka AM Schlumberger M 2016 2015 American Thyroid Association management guidelines for adult patients with thyroid nodules and differentiated thyroid cancer: the American Thyroid Association guidelines task force on thyroid nodules and differentiated thyroid cancer. Thyroid 26 1–133. 26462967 10.1089/thy.2015.0020 PMC4739132

[bib26] Hermsen IG Haak HR De Krijger RR Kerkhofs TM Feelders RA De Herder WW Wilmink H Smit JW Gelderblom H De Miranda NF 2013 Mutational analyses of epidermal growth factor receptor and downstream pathways in adrenocortical carcinoma. European Journal of Endocrinology 169 51–58. (10.1530/EJE-13-0093) 23585556

[bib27] Heuckmann JM Balke-Want H Malchers F Peifer M Sos ML Koker M Meder L Lovly CM Heukamp LC Pao W 2012 Differential protein stability and ALK inhibitor sensitivity of EML4-ALK fusion variants. Clinical Cancer Research 18 4682–4690. (10.1158/1078-0432.CCR-11-3260) 22912387

[bib28] Hou H Sun D Liu K Jiang M Liu D Zhu J Zhou N Cong J Zhang X 2019 The safety and serious adverse events of approved ALK inhibitors in malignancies: a meta-analysis. Cancer Management and Research 11 4109–4118. (10.2147/CMAR.S190098) 31190983 PMC6511621

[bib29] Hrustanovic G Olivas V Pazarentzos E Tulpule A Asthana S Blakely CM Okimoto RA Lin L Neel DS Sabnis A 2015 RAS-MAPK dependence underlies a rational polytherapy strategy in EML4-ALK-positive lung cancer. Nature Medicine 21 1038–1047. (10.1038/nm.3930) PMC4734742 26301689

[bib30] Jung Y Kim P Jung Y Keum J Kim SN Choi YS Do IG Lee J Choi SJ Kim S 2012 Discovery of ALK-PTPN3 gene fusion from human non-small cell lung carcinoma cell line using next generation RNA sequencing. Genes Chromosomes Cancer 51 590–597. 22334442 10.1002/gcc.21945

[bib31] Kelly LM Barila G Liu P Evdokimova VN Trivedi S Panebianco F Gandhi M Carty SE Hodak SP Luo J 2014 Identification of the transforming STRN-ALK fusion as a potential therapeutic target in the aggressive forms of thyroid cancer. PNAS 111 4233–4238. (10.1073/pnas.1321937111) 24613930 PMC3964116

[bib32] Kohler H Latteyer S Hoenes S Theurer S Liao XH Christoph S Zwanziger D Schulte JH Kero J Undeutsch H 2019 Increased ALK activity induces a poorly differentiated thyroid carcinoma in mice. Thyroid 29 1438–1446. 31526103 10.1089/thy.2018.0526 PMC8935483

[bib33] Koivunen JP Mermel C Zejnullahu K Murphy C Lifshits E Holmes AJ Choi HG Kim J Chiang D Thomas R 2008 EML4-ALK fusion gene and efficacy of an ALK kinase inhibitor in lung cancer. Clinical Cancer Research 14 4275–4283. (10.1158/1078-0432.CCR-08-0168) 18594010 PMC3025451

[bib34] Kopetz S Desai J Chan E Hecht JR O’Dwyer PJ Maru D Morris V Janku F Dasari A Chung W 2015 Phase II pilot study of vemurafenib in patients with metastatic BRAF-mutated colorectal cancer. Journal of Clinical Oncology 33 4032–4038. (10.1200/JCO.2015.63.2497) 26460303 PMC4669589

[bib35] Krumbholz M Woessmann W Zierk J Seniuk D Ceppi P Zimmermann M Singh VK Metzler M Damm-Welk C 2018 Characterization and diagnostic application of genomic NPM-ALK fusion sequences in anaplastic large-cell lymphoma. Oncotarget 9 26543–26555. (10.18632/oncotarget.25489) 29899875 PMC5995187

[bib36] Landa I Ibrahimpasic T Boucai L Sinha R Knauf JA Shah RH Dogan S Ricarte-Filho JC Krishnamoorthy GP Xu B 2016 Genomic and transcriptomic hallmarks of poorly differentiated and anaplastic thyroid cancers. Journal of Clinical Investigation 126 1052–1066. (10.1172/JCI85271) PMC4767360 26878173

[bib37] Lei YY Yang JJ Zhang XC Zhong WZ Zhou Q Tu HY Tian HX Guo WB Yang LL Yan HH 2016 Anaplastic lymphoma kinase variants and the percentage of ALK-positive tumor cells and the efficacy of crizotinib in advanced NSCLC. Clinical Lung Cancer 17 223–231. (10.1016/j.cllc.2015.09.002) 26454342

[bib38] Li Y Zhang T Zhang J Li W Yuan P Xing P Zhang Z Chuai S Li J Ying J 2018 Response to crizotinib in advanced ALK-rearranged non-small cell lung cancers with different ALK-fusion variants. Lung Cancer 118 128–133. (10.1016/j.lungcan.2018.01.026) 29571990

[bib39] Lin JJ Zhu VW Yoda S Yeap BY Schrock AB Dagogo-Jack I Jessop NA Jiang GY Le LP Gowen K 2018 Impact of EML4-ALK variant on resistance mechanisms and clinical outcomes in ALK-positive lung cancer. Journal of Clinical Oncology 36 1199–1206. (10.1200/JCO.2017.76.2294) 29373100 PMC5903999

[bib40] Lin YT Liu YN Shih JY 2019 The impact of clinical factors, ALK fusion variants, and BIM polymorphism on crizotinib-treated advanced EML4-ALK rearranged non-small cell lung cancer. Frontiers in Oncology 9 880. (10.3389/fonc.2019.00880) PMC6768009 31608224

[bib41] Mano H 2008 Non-solid oncogenes in solid tumors: EML4-ALK fusion genes in lung cancer. Cancer Science 99 2349–2355. (10.1111/j.1349-7006.2008.00972.x) 19032370 PMC11158085

[bib42] Mano H 2015 The EML4-ALK oncogene: targeting an essential growth driver in human cancer. Proceedings of the Japan Academy: Series B, Physical and Biological Sciences 91 193–201. (10.2183/pjab.91.193) PMC4561238 25971657

[bib43] Matson DR Hardin H Buehler D Lloyd RV 2017 AKT activity is elevated in aggressive thyroid neoplasms where it promotes proliferation and invasion. Experimental and Molecular Pathology 103 288–293. (10.1016/j.yexmp.2017.11.009) 29169802

[bib44] McCusker MG Russo A Scilla KA Mehra R Rolfo C 2019 How I treat ALK-positive non-small cell lung cancer. ESMO Open 4 e000524. (10.1136/esmoopen-2019-000524) PMC6677959 31423342

[bib45] Melillo RM Castellone MD Guarino V De Falco V Cirafici AM Salvatore G Caiazzo F Basolo F Giannini R Kruhoffer M 2005 The RET/PTC-RAS-BRAF linear signaling cascade mediates the motile and mitogenic phenotype of thyroid cancer cells. Journal of Clinical Investigation 115 1068–1081. (10.1172/JCI22758) PMC1062891 15761501

[bib46] Mitiushkina NV Tiurin VI Iyevleva AG Kholmatov MM Filippova EA Moiseyenko FV Levchenko NE Sardaryan IS Odintsova SV Lozhkina AM 2018 Variability in lung cancer response to ALK inhibitors cannot be explained by the diversity of ALK fusion variants. Biochimie 154 19–24. (10.1016/j.biochi.2018.07.018) 30071258

[bib47] Miyakawa M Tsushima T Murakami H Wakai K Isozaki O Takano K 2003 Increased expression of phosphorylated p70S6 kinase and Akt in papillary thyroid cancer tissues. Endocrine Journal 50 77–83. (10.1507/endocrj.50.77) 12733712

[bib48] Nikiforov YE 2011 Molecular analysis of thyroid tumors. Modern Pathology 24 (Supplement 2) S34–S43. (10.1038/modpathol.2010.167) 21455199

[bib49] Ohta K Pang XP Berg L Hershman JM 1997 Growth inhibition of new human thyroid carcinoma cell lines by activation of adenylate cyclase through the beta-adrenergic receptor. Journal of Clinical Endocrinology and Metabolism 82 2633–2638. (10.1210/jcem.82.8.4136) 9253346

[bib50] Panebianco F Nikitski AV Nikiforova MN Kaya C Yip L Condello V Wald AI Nikiforov YE Chiosea SI 2019 Characterization of thyroid cancer driven by known and novel ALK fusions. Endocrine-Related Cancer 26 803–814. (10.1530/ERC-19-0325) 31539879 PMC7002208

[bib51] Penna GC Vaisman F Vaisman M Sobrinho-Simoes M Soares P 2016 Molecular markers involved in tumorigenesis of thyroid carcinoma: focus on aggressive histotypes. Cytogenetic and Genome Research 150 194–207. (10.1159/000456576) 28231576

[bib52] Phelps RM Johnson BE Ihde DC Gazdar AF Carbone DP Mcclintock PR Linnoila RI Matthews MJ Bunn Jr PA Carney D 1996 NCI-Navy Medical Oncology Branch cell line data base. Journal of Cellular Biochemistry: Supplement 24 32–91. (10.1002/jcb.240630505) 8806092

[bib53] Pozdeyev N Gay LM Sokol ES Hartmaier R Deaver KE Davis S French JD Borre PV Labarbera DV Tan AC 2018 Genetic analysis of 779 advanced differentiated and anaplastic thyroid cancers. Clinical Cancer Research 24 3059–3068. (10.1158/1078-0432.CCR-18-0373) 29615459 PMC6030480

[bib54] Priestley P Baber J Lolkema MP Steeghs N De Bruijn E Shale C Duyvesteyn K Haidari S Van Hoeck A Onstenk W 2019 Pan-cancer whole-genome analyses of metastatic solid tumours. Nature 575 210–216. (10.1038/s41586-019-1689-y) 31645765 PMC6872491

[bib55] Recondo G Facchinetti F Olaussen KA Besse B Friboulet L 2018 Making the first move in EGFR-driven or ALK-driven NSCLC: first-generation or next-generation TKI? Nature Reviews: Clinical Oncology 15 694–708. (10.1038/s41571-018-0081-4) 30108370

[bib56] Ricarte-Filho JC Ryder M Chitale DA Rivera M Heguy A Ladanyi M Janakiraman M Solit D Knauf JA Tuttle RM 2009 Mutational profile of advanced primary and metastatic radioactive iodine-refractory thyroid cancers reveals distinct pathogenetic roles for BRAF, PIK3CA, and AKT1. Cancer Research 69 4885–4893. (10.1158/0008-5472.CAN-09-0727) 19487299 PMC2690720

[bib57] Sabir SR Yeoh S Jackson G Bayliss R 2017 EML4-ALK variants: biological and molecular properties, and the implications for patients. Cancers 9 118. (10.3390/cancers9090118) PMC5615333 28872581

[bib58] Schlumberger M Tahara M Wirth LJ Robinson B Brose MS Elisei R Habra MA Newbold K Shah MH Hoff AO 2015 Lenvatinib versus placebo in radioiodine-refractory thyroid cancer. New England Journal of Medicine 372 621–630. (10.1056/NEJMoa1406470) 25671254

[bib59] Schweppe RE Klopper JP Korch C Pugazhenthi U Benezra M Knauf JA Fagin JA Marlow LA Copland JA Smallridge RC 2008 Deoxyribonucleic acid profiling analysis of 40 human thyroid cancer cell lines reveals cross-contamination resulting in cell line redundancy and misidentification. Journal of Clinical Endocrinology and Metabolism 93 4331–4341. (10.1210/jc.2008-1102) 18713817 PMC2582569

[bib60] Seo S Woo CG Lee DH Choi J 2017 The clinical impact of an EML4-ALK variant on survival following crizotinib treatment in patients with advanced ALK-rearranged non-small-cell lung cancer. Annals of Oncology 28 1667–1668. (10.1093/annonc/mdx185) 28407036

[bib61] Siegel RL Miller KD Jemal A 2019 Cancer statistics, 2019. CA: A Cancer Journal for Clinicians 69 7–34. (10.3322/caac.21551) 30620402

[bib62] Soda M Choi YL Enomoto M Takada S Yamashita Y Ishikawa S Fujiwara S Watanabe H Kurashina K Hatanaka H 2007 Identification of the transforming EML4-ALK fusion gene in non-small-cell lung cancer. Nature 448 561–566. (10.1038/nature05945) 17625570

[bib63] Su Y Long X Song Y Chen P Li S Yang H Wu P Wang Y Bing Z Cao Z 2019 Distribution of ALK fusion variants and correlation with clinical outcomes in Chinese patients with non-small cell lung cancer treated with crizotinib. Targeted Oncology 14 159–168. (10.1007/s11523-019-00631-x) 30895431

[bib64] Takezawa K Okamoto I Nishio K Janne PA Nakagawa K 2011 Role of ERK-BIM and STAT3-survivin signaling pathways in ALK inhibitor-induced apoptosis in EML4-ALK-positive lung cancer. Clinical Cancer Research 17 2140–2148. (10.1158/1078-0432.CCR-10-2798) 21415216

[bib65] Tavares C Eloy C Melo M Gaspar Da Rocha A Pestana A Batista R Bueno Ferreira L Rios E Sobrinho Simoes M Soares P 2018 mTOR pathway in papillary thyroid carcinoma: different contributions of mTORC1 and mTORC2 complexes for tumor behavior and SLC5A5 mRNA expression. International Journal of Molecular Sciences 19 1448. (10.3390/ijms19051448) PMC5983778 29757257

[bib66] van der Tuin K Ventayol Garcia M Corver WE Khalifa MN Ruano Neto D Corssmit EPM Hes FJ Links TP Smit JWA Plantinga TS 2019 Targetable gene fusions identified in radioactive iodine-refractory advanced thyroid carcinoma. European Journal of Endocrinology 180 235–241. (10.1530/EJE-18-0653) 30668525

[bib67] van der Velden DL Hoes LR Van Der Wijngaart H Van Berge Henegouwen JM Van Werkhoven E Roepman P Schilsky RL De Leng WWJ Huitema ADR Nuijen B 2019 The drug rediscovery protocol facilitates the expanded use of existing anticancer drugs. Nature 574 127–131. (10.1038/s41586-019-1600-x) 31570881

[bib68] van Eijk R Stevens L Morreau H Van Wezel T 2013 Assessment of a fully automated high-throughput DNA extraction method from formalin-fixed, paraffin-embedded tissue for KRAS, and BRAF somatic mutation analysis. Experimental and Molecular Pathology 94 121–125. (10.1016/j.yexmp.2012.06.004) 22750048

[bib69] van Kuppeveld FJ Van Der Logt JT Angulo AF Van Zoest MJ Quint WG Niesters HG Galama JM Melchers WJ 1993 Genus- and species-specific identification of mycoplasmas by 16S rRNA amplification. Applied and Environmental Microbiology 59 655. (10.1128/AEM.59.2.655-.1993) PMC202165 8434934

[bib70] Vasko V Saji M Hardy E Kruhlak M Larin A Savchenko V Miyakawa M Isozaki O Murakami H Tsushima T 2004 Akt activation and localisation correlate with tumour invasion and oncogene expression in thyroid cancer. Journal of Medical Genetics 41 161–170. (10.1136/jmg.2003.015339) 14985374 PMC1735712

[bib71] Woo CG Seo S Kim SW Jang SJ Park KS Song JY Lee B Richards MW Bayliss R Lee DH 2017 Differential protein stability and clinical responses of EML4-ALK fusion variants to various ALK inhibitors in advanced ALK-rearranged non-small cell lung cancer. Annals of Oncology 28 791–797. (10.1093/annonc/mdw693) 28039177

[bib72] Wood AC Krytska K Ryles HT Infarinato NR Sano R Hansel TD Hart LS King FJ Smith TR Ainscow E 2017 Dual ALK and CDK4/6 inhibition demonstrates synergy against neuroblastoma. Clinical Cancer Research 23 2856–2868. (10.1158/1078-0432.CCR-16-1114) 27986745 PMC5457336

[bib73] Xing M 2013 Molecular pathogenesis and mechanisms of thyroid cancer. Nature Reviews: Cancer 13 184–199. (10.1038/nrc3431) 23429735 PMC3791171

[bib74] Yarchoan M Ma C Troxel AB Stopenski SJ Tang W Cohen AB Pappas-Paxinos M Johnson BA Chen EY Feldman MD 2016 pAKT expression and response to sorafenib in differentiated thyroid cancer. Hormones and Cancer 7 188–195. (10.1007/s12672-016-0253-6) 26994002 PMC10355872

[bib75] Yoshida T Oya Y Tanaka K Shimizu J Horio Y Kuroda H Sakao Y Hida T Yatabe Y 2016 Differential crizotinib response duration Among ALK fusion variants in ALK-positive non-small-cell lung cancer. Journal of Clinical Oncology 34 3383–3389. (10.1200/JCO.2015.65.8732) 27354483

[bib76] Yu ST Ge JN Luo JY Wei ZG Sun BH Lei ST 2019 Treatment-related adverse effects with TKIs in patients with advanced or radioiodine refractory differentiated thyroid carcinoma: a systematic review and meta-analysis. Cancer Management and Research 11 1525–1532. (10.2147/CMAR.S191499) 30863162 PMC6388981

[bib77] Zhang I Zaorsky NG Palmer JD Mehra R Lu B 2015 Targeting brain metastases in ALK-rearranged non-small-cell lung cancer. Lancet: Oncology 16 e510–e521. (10.1016/S1470-2045(15)00013-3) 26433824

[bib79] Zou HY Friboulet L Kodack DP Engstrom LD Li Q West M Tang RW Wang H Tsaparikos K Wang J 2015 PF-06463922, an ALK/ROS1 inhibitor, overcomes resistance to first and second generation ALK inhibitors in preclinical models. Cancer Cell 28 70–81. (10.1016/j.ccell.2015.05.010) 26144315 PMC4504786

