# Supplementary material for: Targeting EML4-ALK gene fusion variant 3 in thyroid cancer
Source: Endocr Relat Cancer. 2021 Apr 20;28(6):377–89. doi: 10.1530/ERC-20-0436 (PMC8183637; doi:10.1530/ERC-20-0436)
Supplement: Supplemental Figure S1 [file supplementary_figure_1.pdf]

Supplementary

Targeting *EML4-ALK* gene fusion variant 3 in thyroid cancer

M.D. Aydemirli[1,2], J.D.H. van Eendenburg[1], T. van Wezel[1], J. Oosting[1], W.E. Corver[1], E. Kapiteijn[2] *, H. Morreau[1] *

[1]Department of Pathology, [2]Department of Medical Oncology, Leiden University Medical Center, 2333 ZA Leiden, The Netherlands.

*Shared last authors

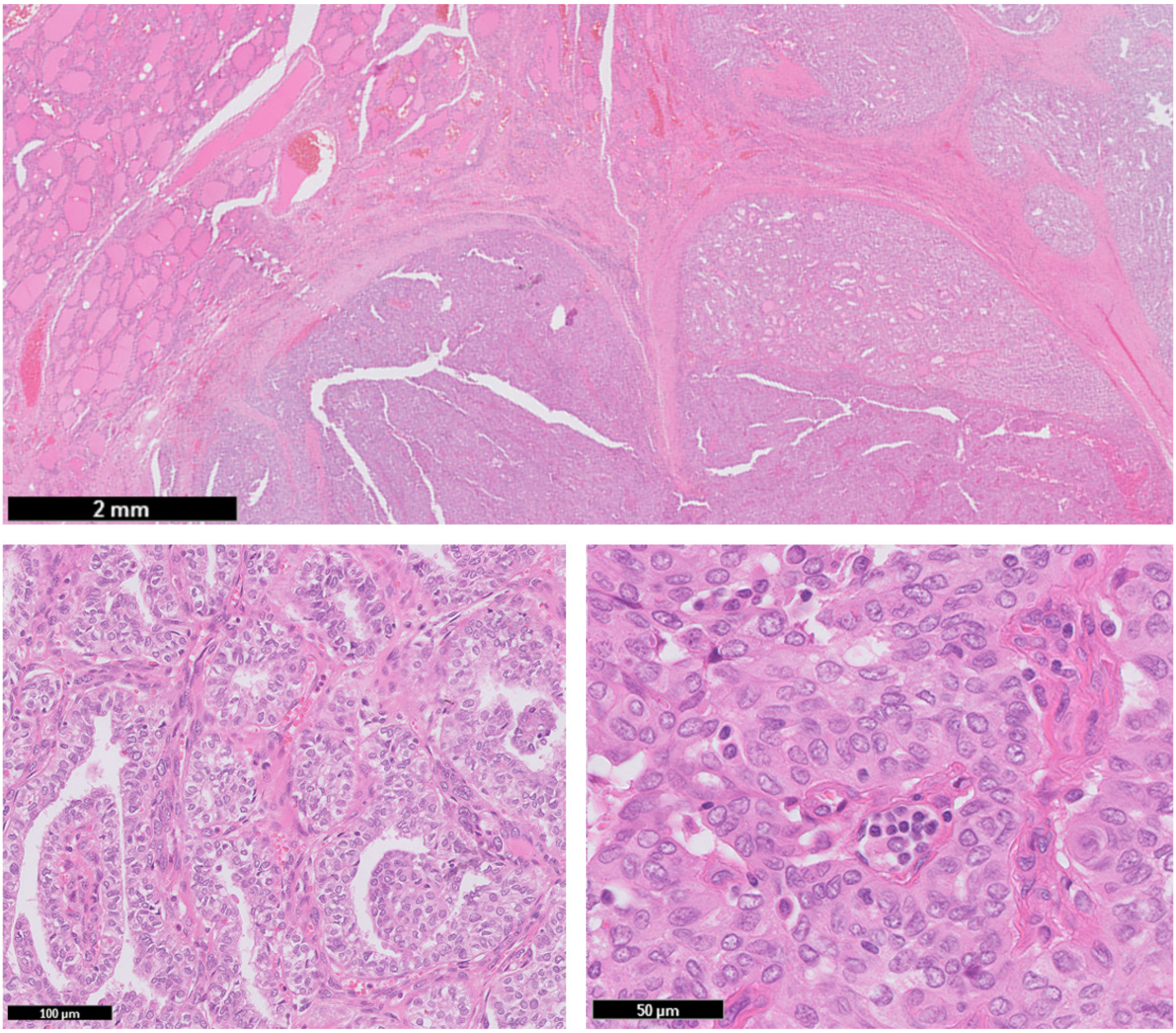

Supplemental Figure S1. Histologic photomicrographs of the primary thyroid lesion (Haematoxylin & Eosin stain). Different components of the resected thyroid tissue including normal thyroid architecture, classic PTC and poorly differentiated areas are shown on low magnification (upper image). Classic papillary thyroid carcinoma component shown on higher magnification (lower left image). Poorly differentiated component on higher magnification (lower right image).
