# Supplementary material for: Targeting EML4-ALK gene fusion variant 3 in thyroid cancer
Source: Endocr Relat Cancer. 2021 Apr 20;28(6):377–89. doi: 10.1530/ERC-20-0436 (PMC8183637; doi:10.1530/ERC-20-0436)
Supplement: Supplemental Figure S2 [file supplementary_figure_2.pdf]

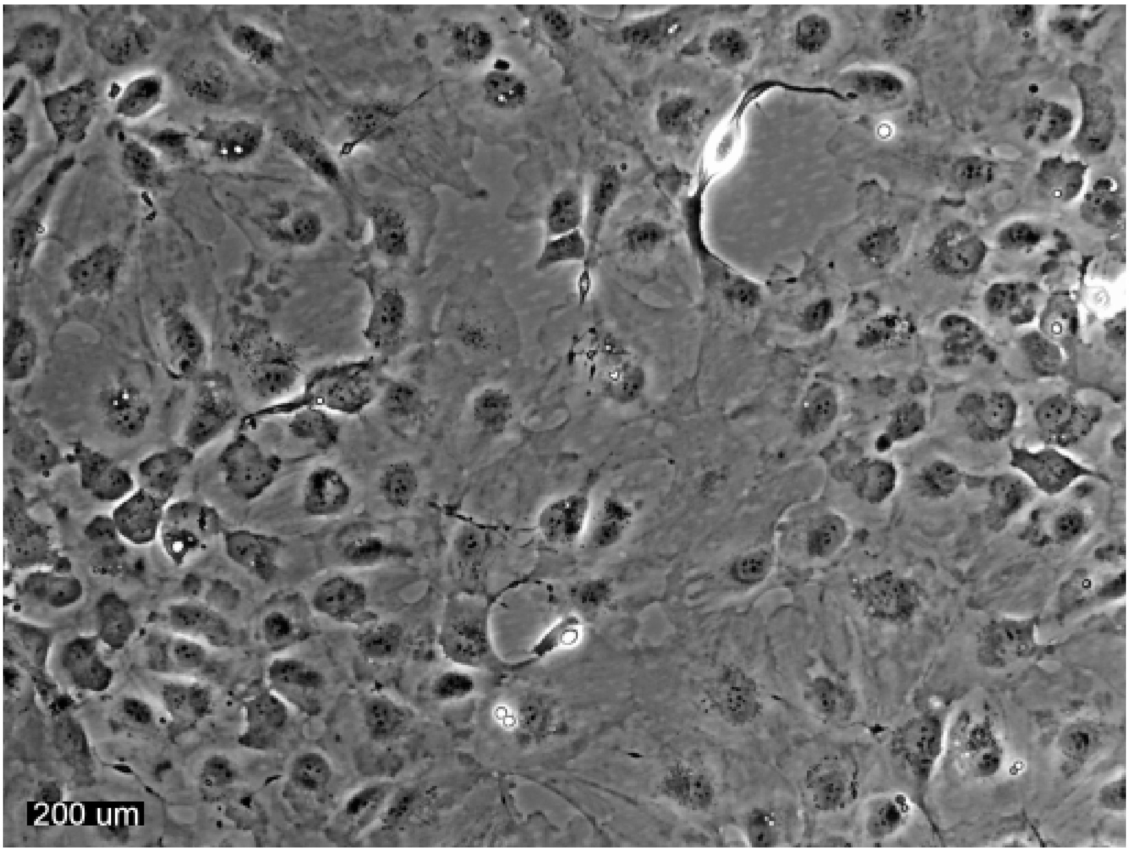

Supplemental Figure S2. Example of cancer cell line morphology of JVE404 p27. The newly established cancer cell line grows in monolayer. (Images made with phase-contrast microscope, Axiovert 40 C Zeiss, magnification 100x. Capture made using Moticam 3 (3.0 MP) camera and image software Motic Images Plus 2.0 ML)
