# Supplementary material for: Targeting EML4-ALK gene fusion variant 3 in thyroid cancer
Source: Endocr Relat Cancer. 2021 Apr 20;28(6):377–89. doi: 10.1530/ERC-20-0436 (PMC8183637; doi:10.1530/ERC-20-0436)
Supplement: Supplemental Figure S3 [file supplementary_figure_3.pdf]

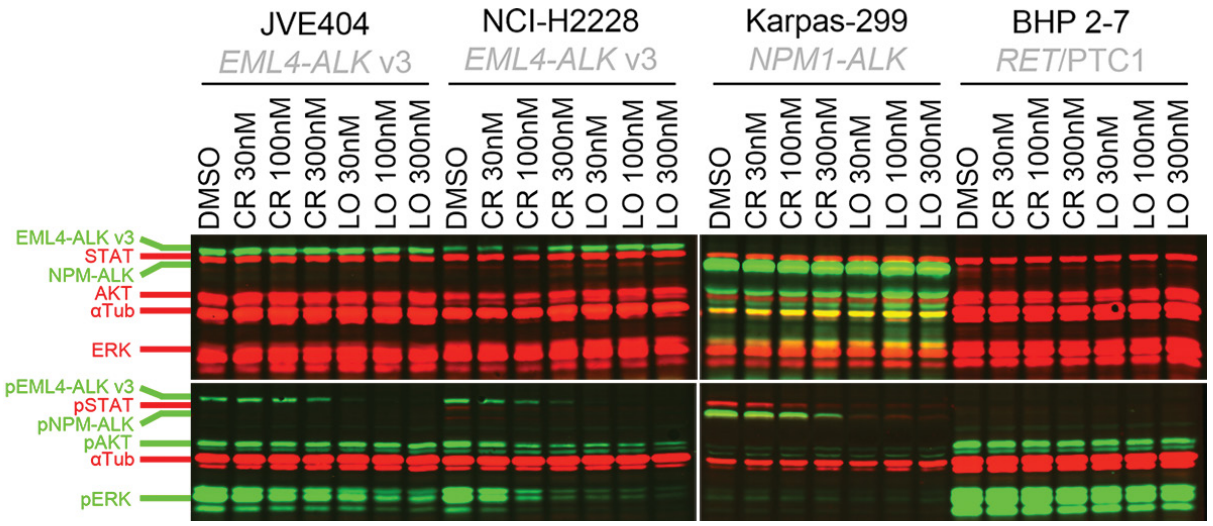

A.

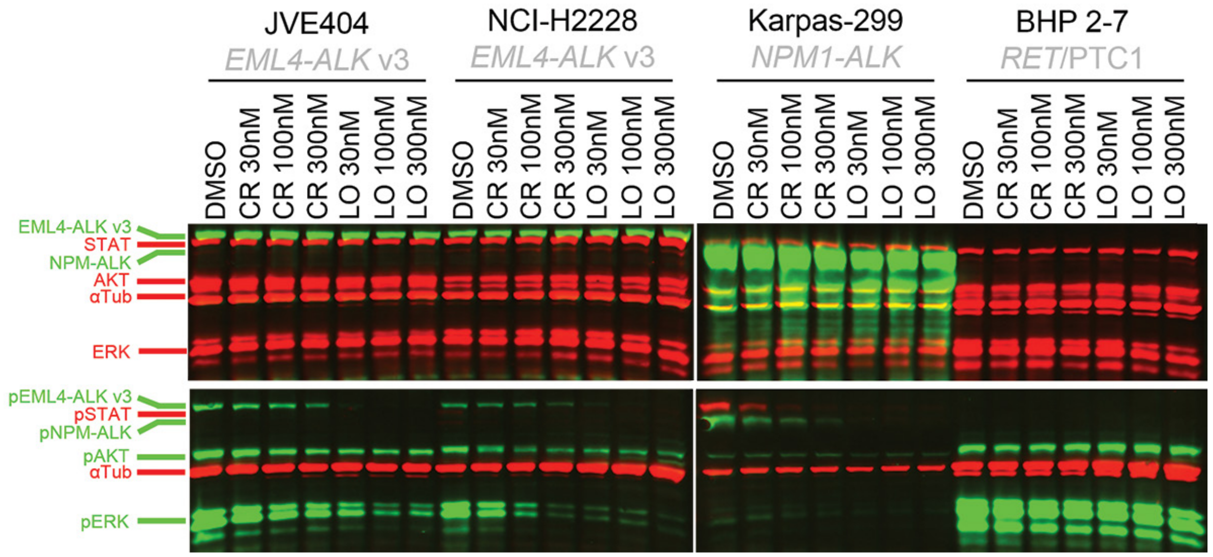

B.

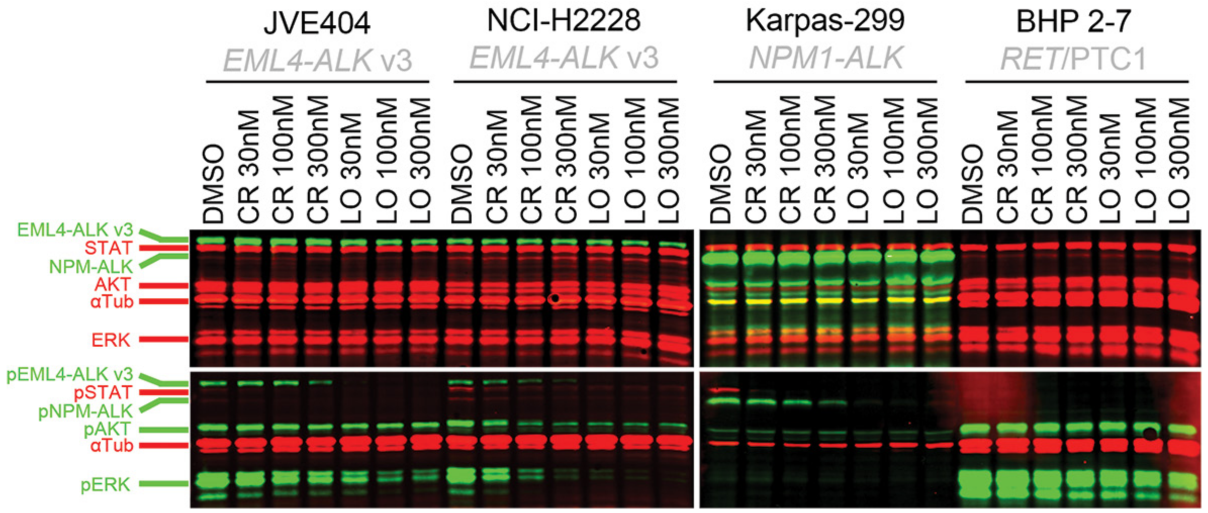

C.

Supplemental Figure S3. Western blots repeated thrice. A: The Western blot shown in the manuscript. B: Western blot repeated. C: Western blot repeated.
