# Supplementary material for: Targeting EML4-ALK gene fusion variant 3 in thyroid cancer
Source: Endocr Relat Cancer. 2021 Apr 20;28(6):377–89. doi: 10.1530/ERC-20-0436 (PMC8183637; doi:10.1530/ERC-20-0436)
Supplement: Supplemental Figure S4 [file supplementary_figure_4.pdf]

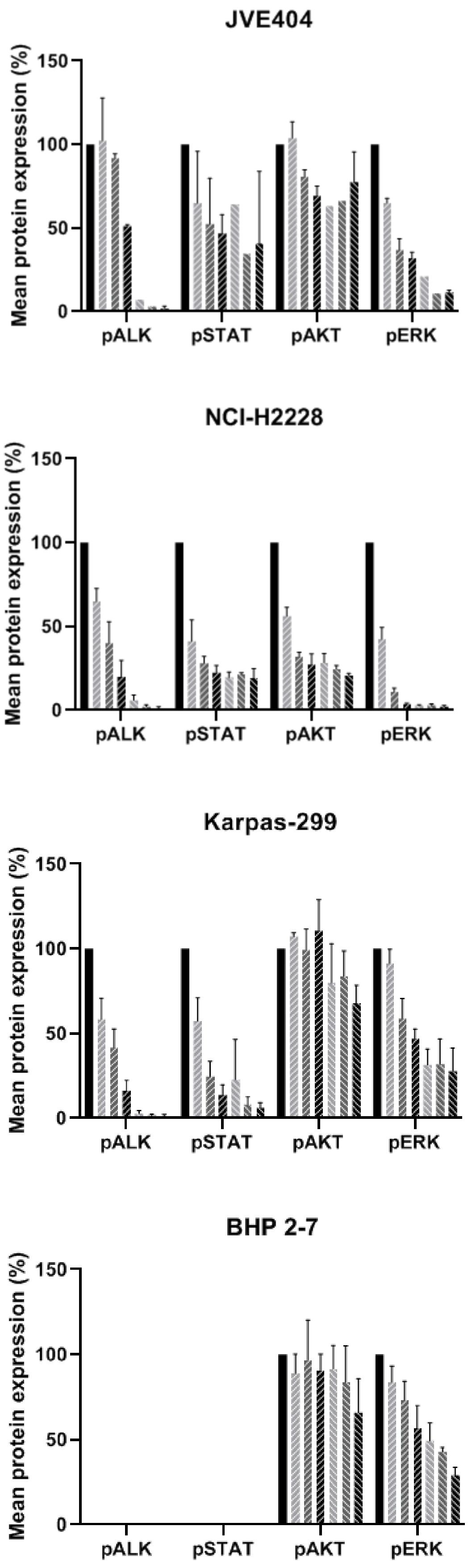

Supplemental Figure S4. Quantification of Western blots shown in Supplemental Figure S3. Normalized mean protein expression in comparison to cells treated with DMSO (every first black bar of the in total seven treatment conditions) observed in cell lines JVE404, NCI-H2228, Karpas-299, BHP 2-7, treated with crizotinib 30 nM, 100 nM, 300 nM (second, third and fourth bar) and lorlatinib 30 nM, 100 nM, 300 nM (fifth, sixth, seventh bar), respectively. The levels of expression of phosphorylated proteins were normalized to household protein control (α-Tubulin). Results are shown as mean protein expression +/– SD of three experiments.
