# Supplementary material for: Targeting EML4-ALK gene fusion variant 3 in thyroid cancer
Source: Endocr Relat Cancer. 2021 Apr 20;28(6):377–89. doi: 10.1530/ERC-20-0436 (PMC8183637; doi:10.1530/ERC-20-0436)
Supplement: Supplemental Figure S5 [file supplementary_figure_5.pdf]

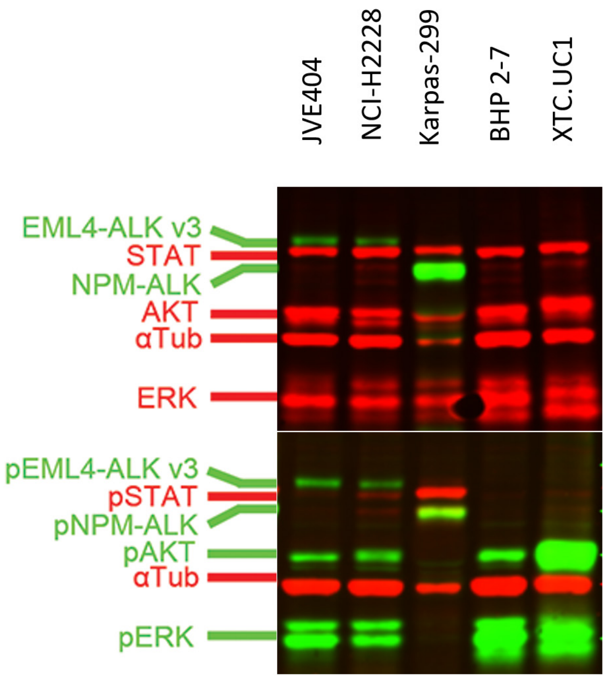

Supplemental Figure S5. Western blot of baseline protein expression. The expression of proteins and phosphorylated proteins (ALK as the fusion protein of EML4 or NPM with conformable molecular sizes; STAT; AKT; ERK and α-Tubulin) for the cancer cell lines JVE404, NCI-H2228, Karpas-299 and BHP 2-7 are shown in DMSO control treated cells. Additionally the thyroid cancer cell line XTC.UC1 has been shown along: XTC.UC1, a Hürthle cell cancer cell line harbouring a near-homozygous genome (Corver et al., 2018), with constitutively activated AKT, as described previously (Aydemirli et al., 2019). XTC.UC1, passage number 122 was kindly provided by Dr. O.H. Clark, UCSF (Zielke et al., 1998).
