# Supplementary material for: Targeting EML4-ALK gene fusion variant 3 in thyroid cancer
Source: Endocr Relat Cancer. 2021 Apr 20;28(6):377–89. doi: 10.1530/ERC-20-0436 (PMC8183637; doi:10.1530/ERC-20-0436)
Supplement: Supplemental Figure S6 [file supplementary_figure_6.pdf]

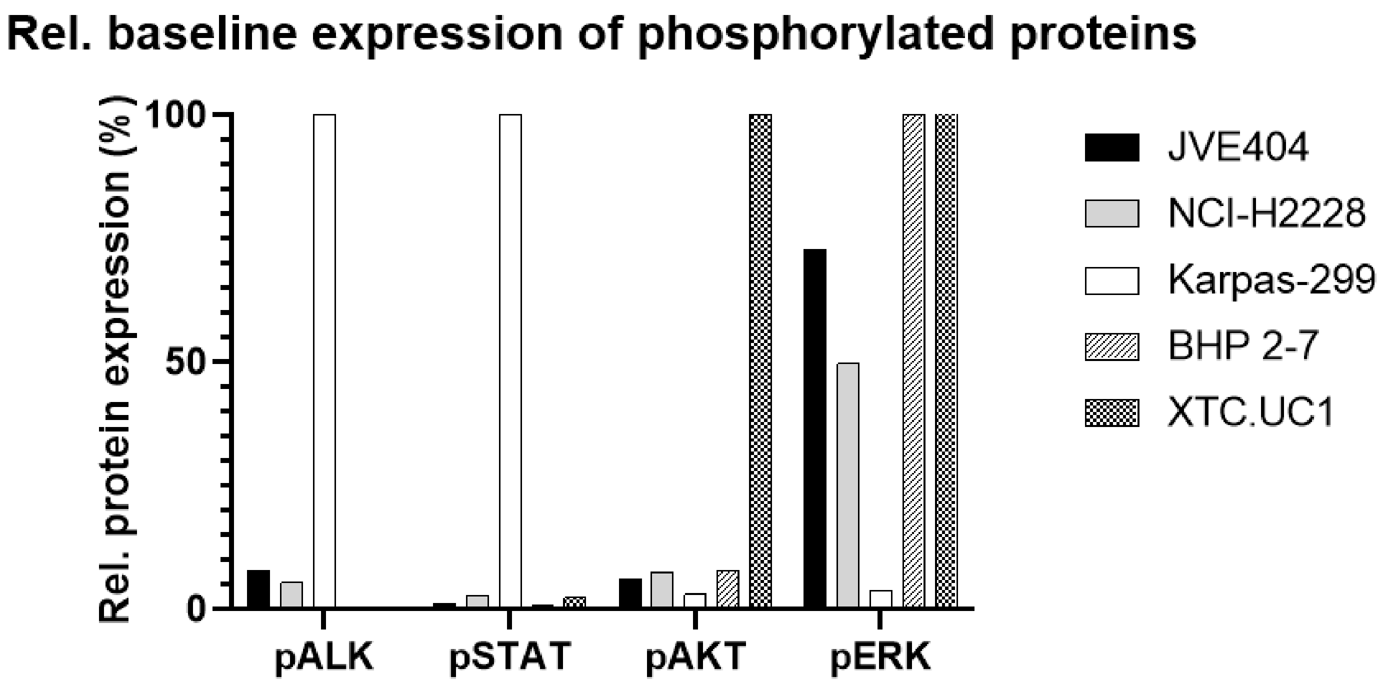

Supplemental Figure S6. Quantification of Western blot shown in Supplemental Figure S5. Protein expression in cells treated with DMSO observed in cell lines JVE404, NCI-H2228, Karpas-299, BHP 2-7 and XTC.UC1. The levels of expression of the phosphorylated proteins were normalized to household protein control (α-Tubulin). Results are shown as percentages of protein expression in the concerning cell line as compared to the cell line with the highest expression level for the corresponding marker (100%). Rel., relative.
